# Supplementary material for: Integrated Glycoproteomics Identifies a Role of N-Glycosylation and Galectin-1 on Myogenesis and Muscle Development
Source: Mol Cell Proteomics. 2020 Dec 19;20:100030. doi: 10.1074/mcp.RA120.002166 (PMC8724610; doi:10.1074/mcp.RA120.002166)
Supplement: Supplemental Figures S1–S4 [file mmc1.pdf]

Supplementary figures

Integrated glycoproteomics identifies a role of *N*-glycosylation and galectin-1 on myogenesis and muscle development

Ronnie Blazev[1], Christopher Ashwood[2,3], Jodie L. Abrahams[2], Long H. Chung[4], Deanne Francis[4], Pengyi Yang[5,6], Kevin I. Watt[1,7], Hongwei Qian[1], Gregory A. Quaife-Ryan[8,9], James E. Hudson[8,9], Paul Gregorevic[1,10,11], Morten Thaysen-Andersen[2], Benjamin L. Parker[1,4*]

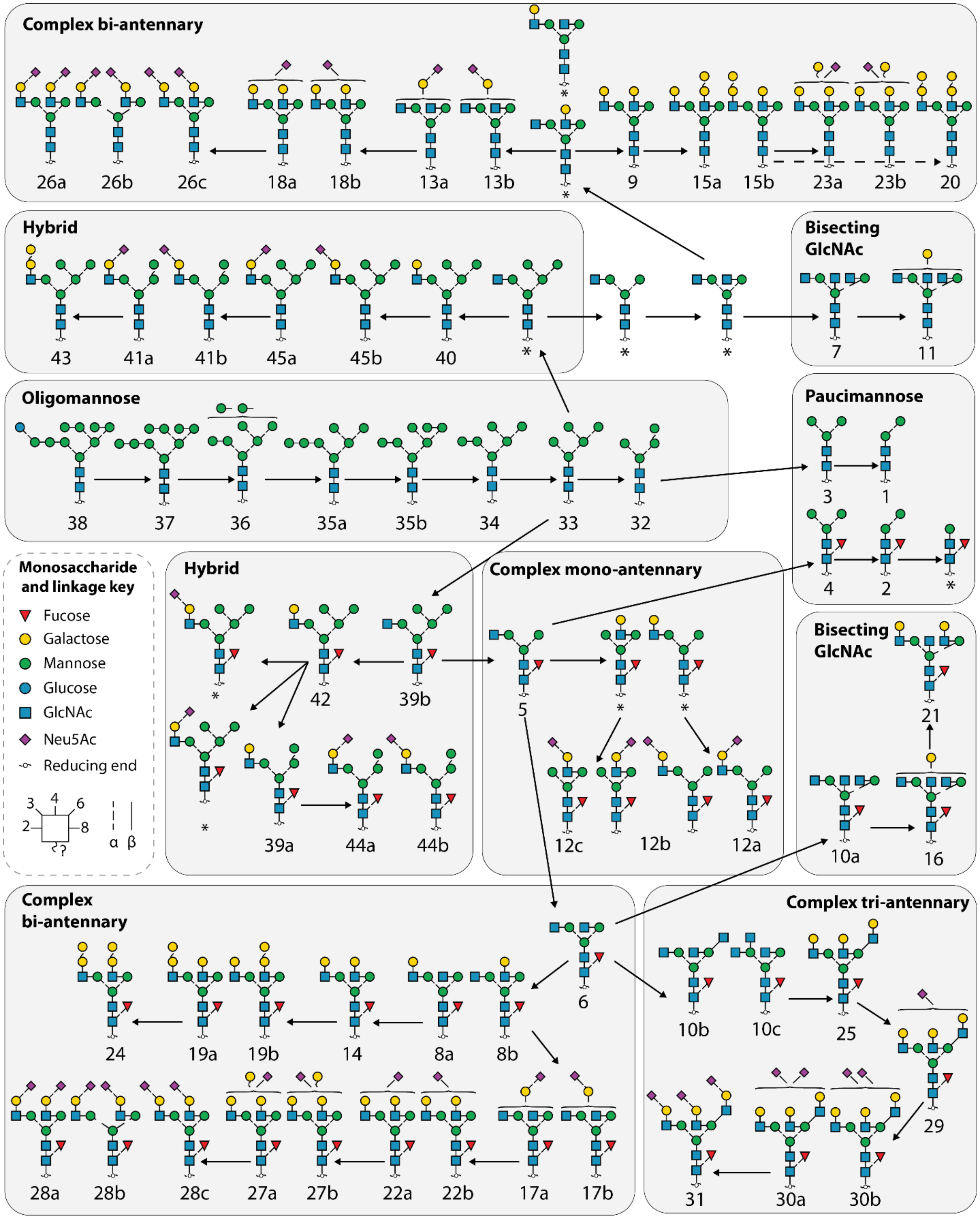

Supplementary Figure S1. Released *N*-glycans from membrane-associated proteins enriched from L6 myoblasts. The glycan identifiers refer to Supplementary Table S2.

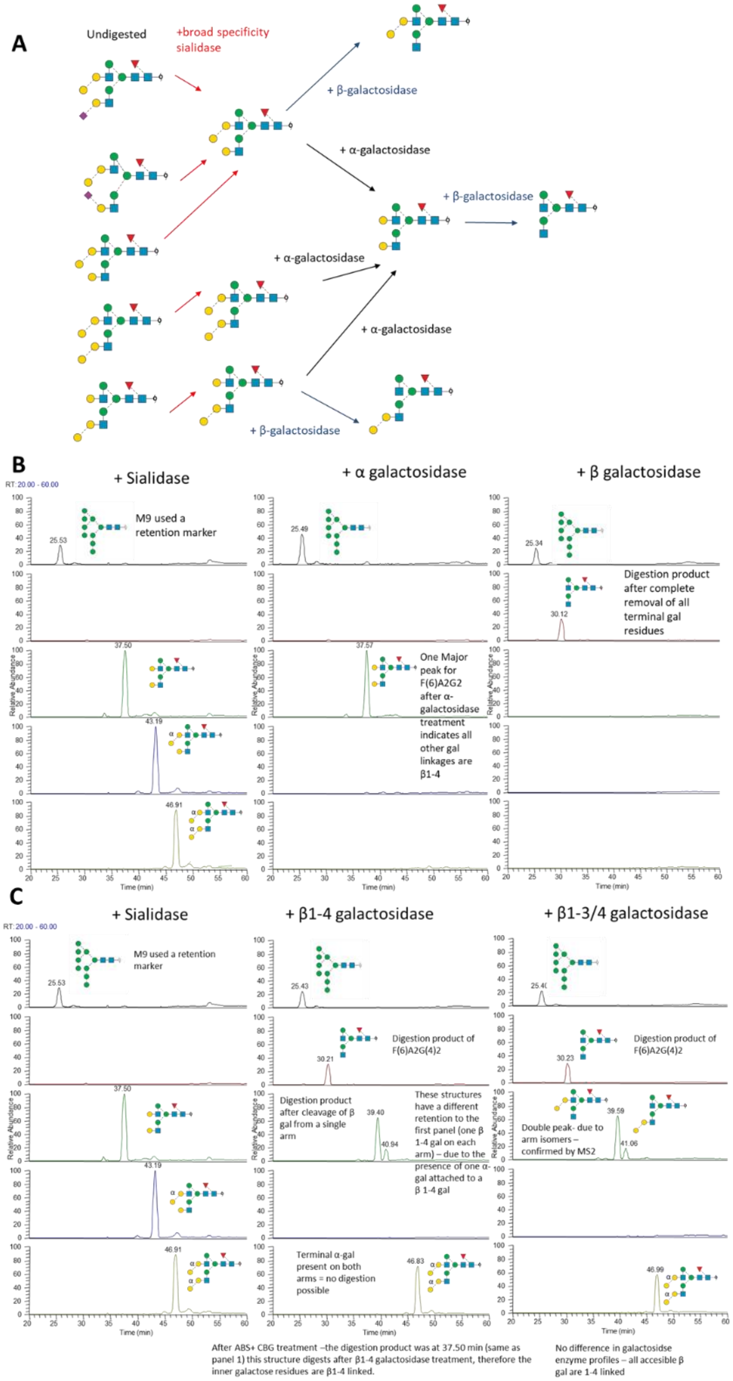

Supplementary Figure S2. Exoglycosidase treatment of released *N*-glycans from membrane-associated proteins enriched from L6 myoblasts. (**A**) Digestion pathways. (**B**) Digestion with broad specificity sialidase, α-galactosidase or β-galactosidase. (**C**) Digestion with broad specificity sialidase, β-1,4-galactosidase or β-1,3/4-galactosidase.

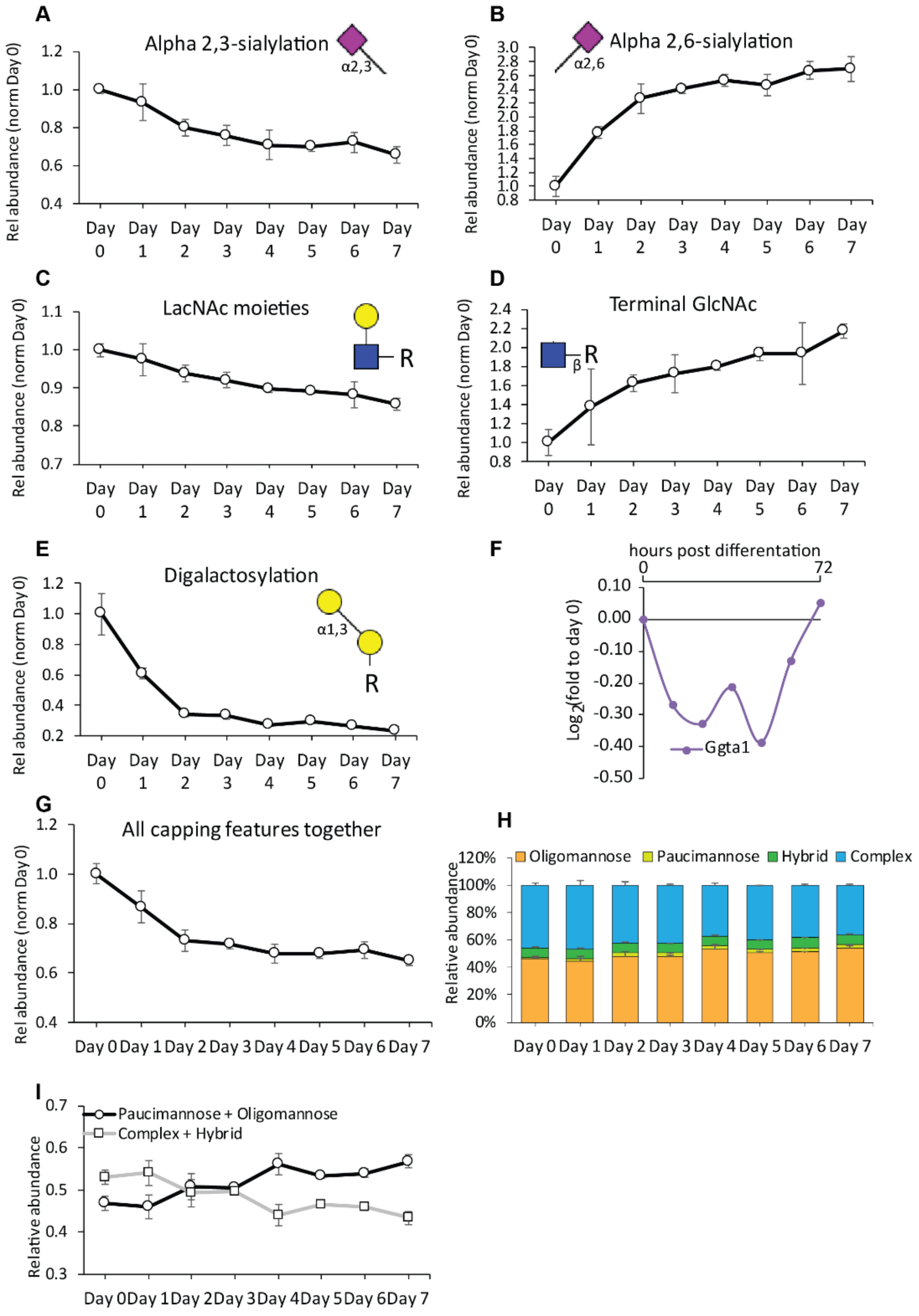

Supplementary Figure S3. Quantification of (**A**) α-2,3-NeuAc-, (**B**) α-2,6-NeuAc-, (**C**) LacNAc-, (**D**) terminal GlcNAc, or (**E**) α-1,3-diGal-containing *N*-glycans. (**F**) Transcriptomic analysis of *Ggta1* during differentiation of C2C12 cells adapted from (37). Quantification of (G) N-glycan capping features, (**H**) distribution of all glycan groups, or (**I**) Pauci- and oligomannose verses complex and hybrid N-glycans.

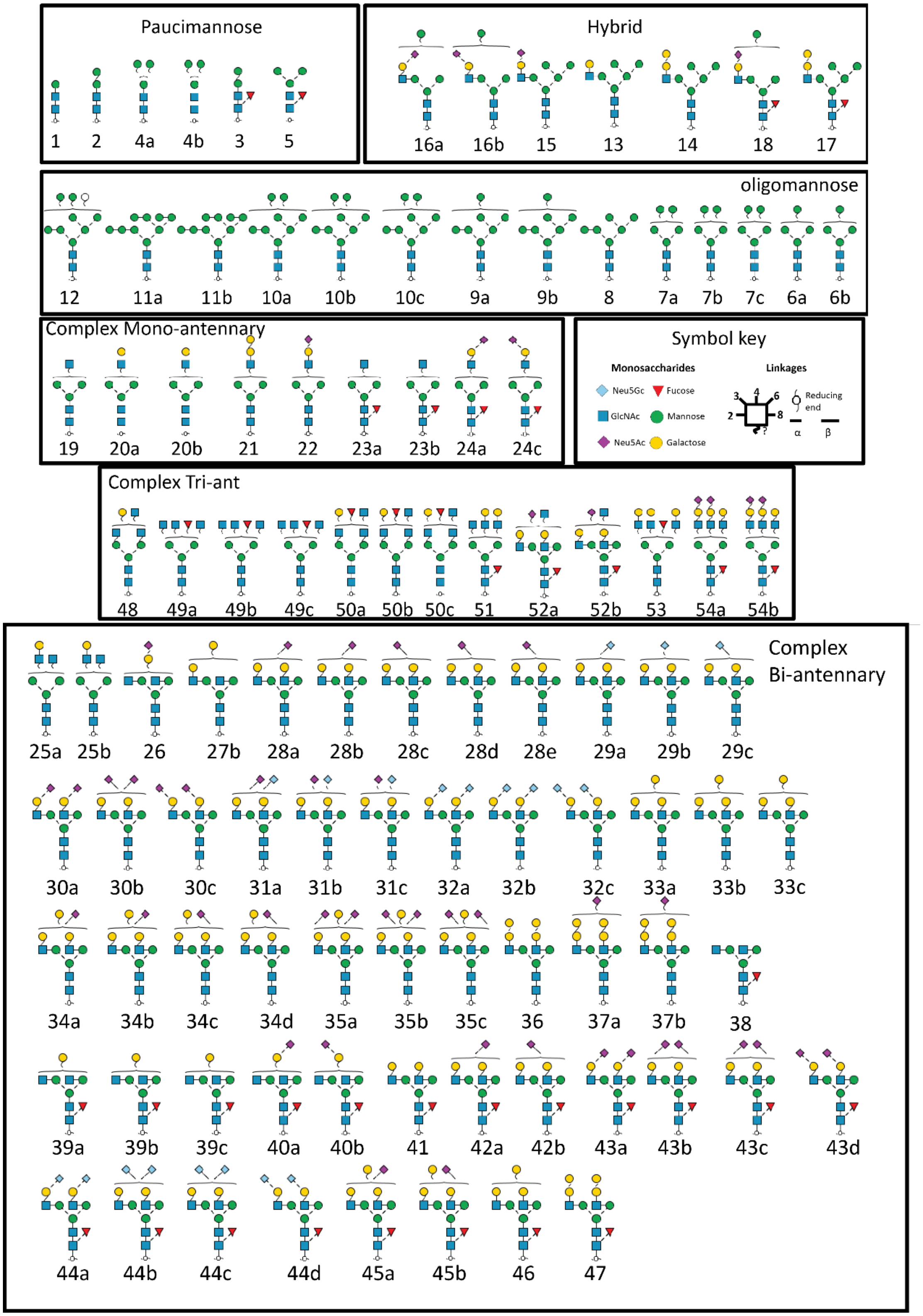

Supplementary Figure S4. Released *N*-glycans from mouse skeletal muscle. Glycan identifiers refer to Supplementary Table S5.
